# Dynamic increase of M2 macrophages is associated with disease progression of colorectal cancers following cetuximab-based treatment

**DOI:** 10.1038/s41598-022-05694-x

**Published:** 2022-01-31

**Authors:** Hyung-Don Kim, Sun Young Kim, Jihun Kim, Jeong Eun Kim, Yong Sang Hong, Buhm Han, Eunyoung Tak, Yeon-Mi Ryu, Sang-Yeob Kim, Tae Won Kim

**Affiliations:** 1 Department of Oncology, Asan Medical Center, University of Ulsan College of Medicine, 88, Olympic-ro 43 gil, Songpa-gu, Seoul, 05505 Republic of Korea; 2 Department of Pathology, Asan Medical Center, University of Ulsan College of Medicine, Seoul, Republic of Korea; 3 Department of BioMedical Sciences, Seoul National University College of Medicine, Seoul, Republic of Korea; 4 Asan Institute for Life Sciences, Asan Medical Center, Seoul, Republic of Korea

**Keywords:** Cancer, Immune evasion, Translational immunology

## Abstract

We aimed to investigate the dynamic changes of gene expression profiles and immune microenvironment linked to resistance to cetuximab-based treatments in patients with metastatic colorectal cancer (mCRC). A total of 106 patients with RAS-wild type mCRC who were treated with cetuximab-based treatments were included as the study population. RNA-sequencing and multiplexed immunohistochemistry were performed using paired or unpaired pre-treatment and post-treatment tumor tissues. Differentially expressed gene analysis of paired pre-treatment and post-treatment tumor tissues that develop acquired resistance (AR) identified the AR signature. Gene ontology analysis of the AR signature indicated enrichment of immune-related pathway genes. Among the immune subsets whose abundance was estimated by CIBERSORT, M2 macrophages showed the most prominent positive correlation with the expression of the AR signature. Among the post-treatment samples, progressive disease (PD) tumors showed a significantly higher abundance of M2 macrophages compared to non-PD tumors. These findings were validated by multiplexed immunohistochemistry analysis: the density of CD68^+^CD206^+^ M2 macrophages significantly increased at the time of PD following cetuximab-based treatment, whereas it did not consistently change in the tumor pairs of non-PD. In conclusion, a dynamic increase of M2 macrophages is associated with disease progression during cetuximab-based treatment of mCRCs. Targeting M2 macrophages is a promising immunotherapeutic strategy in this clinical context.

## Introduction

Colorectal cancer (CRC) is the 3rd most common cancer and the 2nd leading cause of cancer-related deaths, accounting for approximately 1.8 million new cases and 900,000 deaths annually^1^. About one -third of CRC patients ultimately progress to metastatic disease, and patients with metastatic CRC have a 5-year survival rate of only 12%^2^. Despite substantial efforts to improve clinical outcomes, the development of novel treatment strategies with better efficacy is urgently needed for patients with metastatic CRC.

Anti-epidermal growth factor receptor (EGFR) therapies such as cetuximab and panitumumab are the mainstay of molecularly targeted treatment for patients with RAS wild-type metastatic CRCs. These agents inhibit the receptor tyrosine kinases and thus block multiple downstream signaling pathways involved in cell survival, proliferation, metastasis, and angiogenesis^3^. However, most patients ultimately develop resistance to anti-EGFR-based treatments. Several processes such as secondary genetic abnormalities and alterations in angiogenic pathways have been suggested as the mechanisms of acquired resistance (AR). However, no therapeutic approaches have been proven to confer a clinical benefit to overcome the resistance in this clinical context, and efforts are being made to delineate the mechanism of resistance and develop novel therapeutic strategies.

The CRC microenvironment is comprised of heterogeneous immune subsets, which dynamically interact with tumor cells and the stromal component and play important roles in immune evasion and tumor progression^4,5^. Since EGFR is expressed on various immune cells, including myeloid cells^6,7^ and T cell subsets^8^, anti-EGFR treatment may modulate the immune microenvironment of CRC. In addition, cetuximab treatment in combination with chemotherapy has been shown to promote immunogenic cell death, thereby activating anti-tumor immune responses^9^. Among the immune subsets that could potentially play an important role in the context of anti-EGFR treatment is M2 macrophages. M2 macrophages are an immunosuppressive subtype of tumor-associated macrophages (TAMs)^10–15^, which are associated with poor survival outcomes of CRC patients^16,17^. EGFR blockade reduces the production of M2 macrophage-promoting cytokine IGF-1, thereby inhibiting M2 polarization^18^. However, their involvement in the resistance to anti-EGFR-based treatments remains largely unknown.

In this study, using serially collected paired tumor tissue samples, we aimed to investigate the dynamic transcriptomic profiles of CRCs associated with resistance to cetuximab-based treatments. In particular, we focused on the gene signature associated with M2 macrophages and validated our findings by multiplexed immunohistochemistry.

## Results

### Patient characteristics

Baseline characteristics of the study patients are summarized in Table 1. Their median age was 57 (range 20–80 years), and 69 patients (65.1%) were men. About 80% of the patients had a left side tumor and most of the patients (n = 96, 90.6%) had initially metastatic disease (M1 disease) at the time of diagnosis of CRC, while 10 patients (9.4%) had recurrent M1 disease after curative resection of stage III tumors. Among those who had initially resectable disease (n = 10), 7 patients received adjuvant chemotherapy and 3 received neoadjuvant chemoradiation therapy for rectal cancer. There were 89 (84.0%) and 17 (16%) patients who received cetuximab-based treatment as first-line and third-line treatments, respectively. Details of their chemotherapy regimens are presented in Table 1. The median progression-free survival (PFS) of the entire cohort was 13.0 months (95% confidence interval [CI] 12.3–14.2 months) (Supplementary Fig. 1) with patients in the first- and third-line at 13.7 months (95% CI 13.0–14.9 months) and 6.5 months (95% CI 3.5–10.5 months), respectively.

**Table 1:** Clinical characteristics of the study patients.

Variable	Study population (n = 106)
Age (years)	57 (range 22–80)
Male sex	69 (65.1%)
**Primary tumor location**	
Right side	21 (19.8%)
Left side	85 (80.2%)
RAS wild-type	106 (100%)
BRAF wild-type	106 (100%)
**Disease setting**	
Recurrent	10 (9.4%)
Initially metastatic	96 (90.6%)
**Treatment line**	
1st line	89 (84.0%)
3rd line	17 (16.0%)
**Best response**	
Complete response	7 (6.6%)
Partial response	78 (73.6%)
Stable disease	12 (11.3%)
Progressive disease	9 (8.5%)
**Treatment regimen**	
Cetuximab + FOLFIRI*	74 (69.8%)
Cetuximab + FOLFOX^†^	5 (4.7%)
Cetuximab + Irinotecan	12 (11.3%)
Cetuximab alone	15 (14.2%)

*5-fluorouracil plus irinotecan.

^†^5-fluorouracil plus oxaliplatin.

### Gene signature of acquired resistance

**Figure 1:**
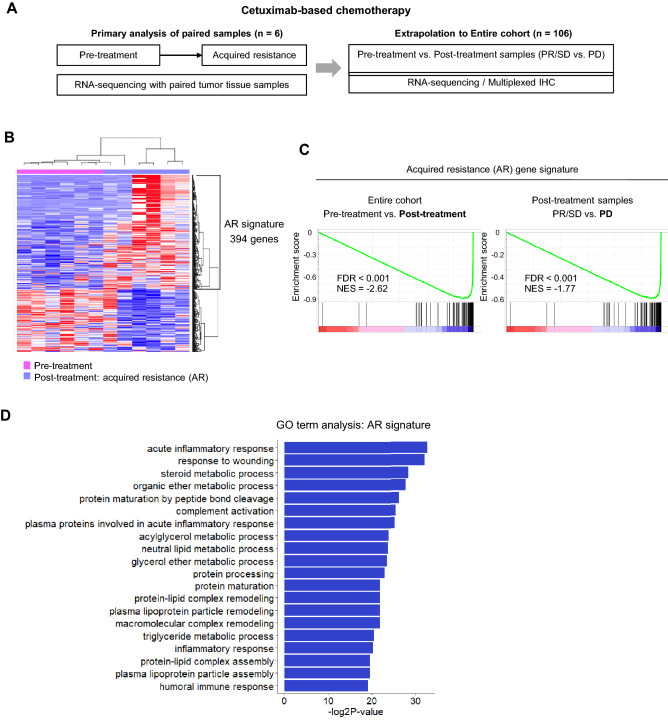
Gene signature of acquired resistance. (**A**) Overall scheme of the study. (**B**) Heatmap of differentially expressed genes between pre-treatment and post-treatment tumor samples in patients who develop acquired resistance (AR) (n = 6 pairs). (**C**) Gene Set Enrichment Analysis of the AR signature in the entire cohort and post-treatment samples. (**D**) Gene ontology pathway analysis of the AR signature.

We first focused our investigation on the gene signature associated with the development of AR during the cetuximab-based treatments. We analyzed the gene expression profiles of patients who had paired pre-treatment baseline tumor tissue and post-treatment tumor tissue that developed AR (n = 6) (Fig. 1A). Differentially expressed gene analysis identified 394 genes specifically up-regulated in post-treatment AR tumor tissue samples, which we defined as the AR signature (Fig. 1B and Supplementary Table 2). We next examined the expression levels of the AR signature in the samples of the entire cohort (Fig. 1A). The AR signature was enriched in the post-treatment samples (vs. the pre-treatment samples) (normalized enrichment score [NES] − 2.62, false discovery rate [FDR] < 0.001) (Fig. 1C). Among the post-treatment samples, the progressive disease (PD) tumor samples showed enrichment for the AR signature compared to the partial response (PR)/stable disease (SD) tumor samples (NES − 1.77, FDR < 0.001) (Fig. 1C). This suggested that the AR signature was prominently expressed in the post-treatment tumors samples, especially in those that progressed during cetuximab-based treatment. Gene ontology term analysis identified several pathways of inflammation and the immune response as the top signals associated with the AR signature (Fig. 1D).

### Association between the acquired resistance signature and M2 macrophages

**Figure 2:**
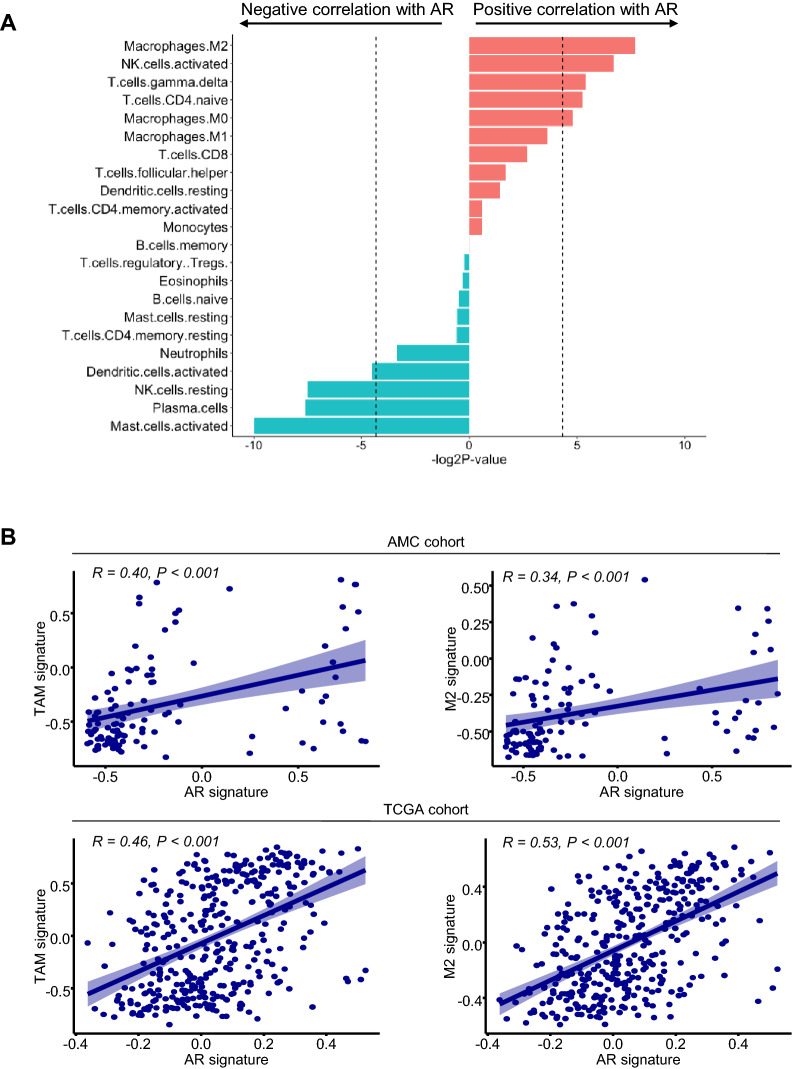
Correlation between the expression levels of the AR signature and M2 macrophage-related parameters. (**A**) Bar graph showing the correlation between the expression levels of the AR signature represented by the enrichment score calculated by Gene Set Variation Analysis (GSVA) and the abundance of immune subsets estimated by CIBERSORT. (**B**) Correlation between the expression levels of the AR gene signature and tumor-associated macrophages and M2 macrophage signatures in the study cohort (upper panel) and TCGA cohort (lower panel).

Given that the AR signature was linked to immune-related signals, we performed immune deconvolution of the transcriptomic data using CIBERSORT. Figure 2A shows the correlation between the fraction of 22 immune subsets and the expression levels of the AR signature, which are represented by the enrichment score. M2 macrophages showed the most prominent positive correlation with the expression levels of the AR signature (*P* = 0.005).

We then examined the relationship between the expression levels of the AR signature and those of the previously reported TAM and M2 signatures^19^. The TAM and M2 signatures showed positive correlations with the AR signature (R = 0.40, *P* < 0.001 and R = 0.34, *P* < 0.001, respectively) (Fig. 2B, upper panel). These findings were validated in the TCGA CRC cohort (R = 0.46, *P* < 0.001 and R = 0.53, *P* < 0.001, respectively) (Fig. 2B, lower panel), supporting an association between the AR and M2-related signatures in CRC.

### Abundance of M2 macrophages according to the clinical settings

**Figure 3:**
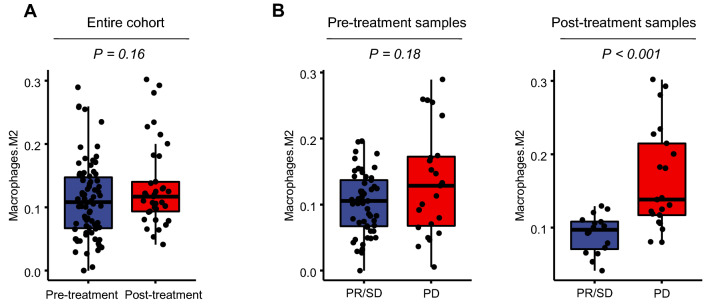
Comparison of the abundance of M2 macrophages estimated by immune deconvolution. The abundance of M2 macrophages estimated by CIBERSORT was compared between pre-treatment versus post-treatment tumor samples in the entire cohort (**A**) and tumors with partial response or stable disease versus those with progressive disease among the post-treatment samples (**B**).

We next compared the abundance of M2 macrophages estimated by CIBERSORT according to the different clinical settings. The abundance of the M2 macrophages was comparable between the overall pre-treatment and post-treatments samples (*P* = 0.16) (Fig. 3A). Among the post-treatment samples, PD tumors showed significantly higher levels of M2 macrophage abundance compared to PR/SD tumors (*P* < 0.001) (Fig. 3B).

### M2 macrophages in paired pre-treatment and post-treatment tumor tissues

**Figure 4:**
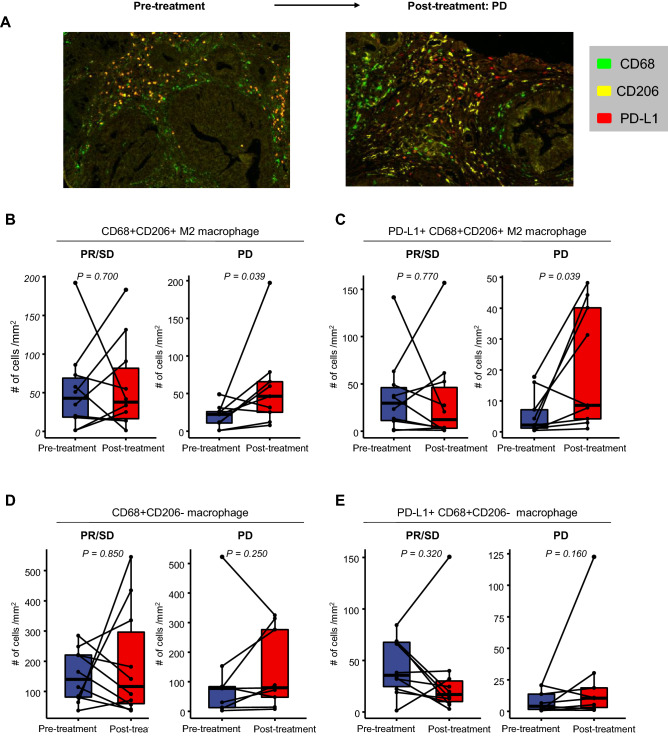
Dynamic changes of the density of the M2 macrophages according to different clinical settings. (**A**) Representative scans of multiplexed immunohistochemistry showing dynamic changes of CD68, CD206 and PD-L1 between pre-treatment tumor and post-treatment tumor with progressive disease. (**B**–**E**) Dynamic changes in the density in the following immune subsets according to the clinical response: (**B**) CD68^+^CD206^+^ M2 macrophages; (**C**) PD-L1^+^ CD68^+^CD206^+^ M2 macrophages; (**D**) CD68^+^CD206^−^ macrophages; and (**E**) PD-L1^+^ CD68^+^CD206^−^ macrophages.

To validate the abundance of M2 macrophages in post-treatment PD tumor samples from the transcriptome data, we performed multiplexed immunohistochemistry using paired pre-treatment and post-treatment tumor tissues (Fig. 4A). Importantly, the density of the CD68^+^CD206^+^ M2 macrophages significantly increased at the time of PD following cetuximab-based treatment (*P* = 0.039), whereas it did not consistently change between the tumor pairs of PR/SD (*P* = 0.700) (Fig. 4B). The density of M2 macrophages expressing PD-L1 also substantially increased in the post-treatment PD tumors (*P* = 0.039), whereas the PR/PD samples did not (*P* = 0.770) (Fig. 4C).

On the other hand, the density of CD68^+^CD206^-^ macrophages did not significantly change during the treatment, regardless of the response (*P* = 0.850 and *P* = 0.250 for PR/SD and PD samples, respectively) (Fig. 4D). The same was true for CD68^+^CD206^-^ macrophages that expressed PD-L1 (*P* = 0.320 and *P* = 0.160 for the PR/SD and PD samples, respectively) (Fig. 4E).

## Discussion

In this study, based on longitudinally collected paired tumor tissue samples from mCRC patients treated with cetuximab-based chemotherapy, we identified a gene signature representing AR from CRC patients treated with cetuximab-based treatments. Based on the unexpected association of the gene signature with immune-related pathways, we focused our investigation on the dynamic immune microenvironment, revealing the enrichment of M2 macrophages estimated by immune deconvolution in post-treatment tumors that progressed during cetuximab-based treatments. We validated this finding using paired tumor tissue samples with different clinical outcomes by multiplexed immunohistochemistry, confirming the increase in the density of M2 macrophages during disease progression. To our knowledge, the current study is the first to delineate a specific association between a dynamic change in the immune microenvironment and the development of resistance to cetuximab-based treatments in patients with CRC, which could have therapeutic implications for developing novel treatment strategies in this clinical setting.

The emergence of secondary mutations or the activation of alternative oncogenic pathways represents the classical mechanism of therapeutic resistance to targeted agents in CRC. Several secondary genetic abnormalities in *KRAS*^20,21^, *MET*^22^, *ERBB2*^23,24^, *IGF-1*^25^ and *NF-1*^26^ have been suggested to be involved in the mechanism of cetuximab resistance. However, no treatment has been proven efficacious to overcome this resistance, and the heterogeneity of secondary genetic abnormalities limits the development of universally applicable therapeutic strategies to overcome the resistance. When considering strategies for developing novel treatment strategies to overcome resistance, immunotherapy has a beneficial aspect of being applicable to most tumors regardless of their genetic changes. Previous transcriptome analyses have reported an altered immune microenvironment of CRC following anti-EGFR treatment and its association with different treatment outcomes^26,27^. In particular, Woolston et al.^26^ reported enhanced T cell infiltration and cytolytic scores in PD samples (vs. baseline samples) in tumors from patients who exhibited a prolonged benefit, which was not observed in the patient subgroup with primary disease progression. However, this may not sufficiently explain the immune evasion mechanism linked to disease progression, given the well-demonstrated protective activity of T-cell infiltrates in CRC^28,29^.

M2 macrophages are a well-known immune subset in the tumor microenvironment, inhibiting anti-tumor immune responses and promoting tumor progression^10,11,13–15^, and the majority of PD-L1 expressing cells within the tumor microenvironment are TAMs and these subsets play a critical role in immune suppression^12,30^. Therefore, our findings showing enrichment of M2 macrophages in PD tumors, especially those expressing PD-L1, provide novel insights into the immune evasion mechanism of tumors associated with resistance to cetuximab-based treatments based on the differential dynamic features of tumors that eventually progress during cetuximab-based treatment and those maintaining anti-tumor activity (i.e., SD or PR). In a preclinical study of colon cancer cells, conditioned media of colon cancer cell lines whose EGFR expression was knocked down led to down-regulation of M2 macrophage-related markers such as IL-10, Arg1, CCL17, CCL22, and IL-4, while up-regulating M1 macrophage-related markers including IL-12, CCR7, and TNF-α at the same time^18^. Similarly, another study of triple-negative breast cancer spheroids showed that cetuximab-conjugated gold nanorods in combination with irradiation reduced expression of the macrophage mannose receptor, which indicates polarization to the M1 anti-tumor phenotype^31^. Together with our findings that resistance to cetuximab-based chemotherapy is associated with the enrichment of M2 macrophage-related signals, we assume that EGFR inhibition may be no longer capable of promoting polarization to M1 nor suppressing M2 TAM-related features in the CRC microenvironment developing resistance to cetuximab-based treatments.

In preclinical models, M2 macrophages have been shown to be implicated in the resistance to chemotherapy^32,33^ via various mechanisms such as the production of immunosuppressive cytokines like IL-10 and TGF-β and tumor-invasion promoting factors and proangiogenic factor VEGF as well as the overexpression of PD-L1^34^. M2 macrophages have also been shown to mediate resistance to sorafenib in hepatocellular carcinoma via secretion of hepatocyte growth factor^35^. In CRC, M2 macrophages conferred resistance to 5-fluorouracil through epithelial-mesenchymal transition, PI3K/AKT pathways and caspase-mediated apoptosis via chemokine CCL22^17^. However, their involvement in resistance to cetuximab-based-treatment has not been specifically investigated previously using actual clinical specimens. In the era of immunotherapy, substantial efforts are being made to develop novel immunotherapies that modulate TAMs and M2 macrophages, which may have implications for overcoming chemoresistance. Various agents with different mechanisms, such as inhibition of the recruitment and survival of TAM and M2 polarization, are currently under clinical development^10,11,15^. In particular, activation of the colony stimulating factor-1 (CSF-1)/colony stimulating factor-1 receptor (CSF-1R) axis promotes polarization of TAMs toward M2 macrophages^11,14,15^, and early phase studies evaluating several CSF-1R inhibitors and anti-CSF-1 antibodies are currently ongoing^10,11,15^. Considering the substantial heterogeneity in the composition of M2 macrophages and the associated immune microenvironment in colorectal cancer^4,5^, it would be important to delineate the specific subgroup of CRC patients and the clinical context in which M2 macrophages are meaningfully enriched. In addition, future development of novel agents targeting TAMs or M2 macrophages should focus on identifying an optimal combination with current standard treatments.

One of the most notable aspects of our study is our primary analysis and validation using multiplexed immunohistochemistry were based on paired pre-treatment and post-treatment samples, which was feasible because of the active multidisciplinary management of metastatic CRC^36^. Paired analysis enabled the evaluation of dynamic changes induced by cetuximab-based treatment among actual patients, revealing a novel signature representing AR. This gene signature will be an important resource for mining additional key genes and pathways involved in the resistance to cetuximab-based treatments. Although previous studies dealing with AR with cetuximab analyzed paired samples, they were limited either by using a small number of samples^27^ or focusing only on PD tumors^26^, which precludes a comparison between post-treatment samples with different responses (i.e., SD/PR vs. PD).

There are some limitations of our study to be discussed. First, the clinical samples were retrospectively collected, and the study patients were heterogeneous, with different lines of therapy and different chemotherapy regimens. Also, since our cohort was selected based on the availability of tumor tissue samples, our cohort may not fully recapitulate that of actual clinical practice. In addition, it was impossible to acquire and analyze post-treatment tumor samples that achieved a radiological CR. On the other hand, since cetuximab was administered together with cytotoxic chemotherapeutic agents in most of the cases and our analyses included a heterogeneous population receiving cetuximab treatments (i.e., different treatment lines and/or regimens), the exact contribution of cetuximab to the increase in M2 macrophages is not fully known. Indeed, M2 macrophages have been suggested to confer resistance to 5-fluorouracil treatment in CRC cells^17^ and cisplatin treatment in gastric cancer cells^37^ via activation of the PI3K/AKT signaling pathway. Nevertheless, given that a cetuximab-chemotherapy combination is the standard treatment in this clinical context, our results may provide practical insights into understanding the mechanism of resistance and developing novel therapeutic strategies. In addition, whether the association between the development of therapeutic resistance and an increase in M2 macrophages is specific to cetuximab-based treatments remains uncertain.

In conclusion, a dynamic increase of M2 macrophages is associated with disease progression during cetuximab-based treatments for metastatic CRCs. Future studies should focus on the identification of specific therapeutic targets involved in promoting M2 polarization and the development of novel immunotherapeutic strategies in this clinical context.

## Methods

### Study patients and analysis of the tumor tissue specimens

A total of 106 patients with RAS/BRAF wild-type metastatic CRC who were treated with cetuximab-based treatments as their 1st line or 3rd line treatment at Asan Medical Center (Seoul, Korea) between May 2011 and September 2018 were included as the study population. Tumor responses were assessed every 6–8 weeks according to the response evaluation criteria in solid tumors (RECIST) version 1.1.

Pre-treatment and post-treatment tumor tissue samples were obtained from biopsy of the primary or metastatic site or tissue obtained during palliative surgery (Fig. 1A and Supplementary Table 1). Paired pre-treatment baseline and post-treatment samples were obtained from 35 (33.0%) patients, while 55 (51.9%) and 16 (15.1%) had pre-treatment and post-treatment samples only, respectively.

The Institutional Review Board of Asan Medical Center approved the study protocol, and informed consent was obtained from all participants. All procedures followed were in accordance with the ethical standards of the responsible committee on human experimentation (institutional and national) and with the Helsinki Declaration of 1964 and its later versions.

### Definition of acquired resistance

AR was defined as disease progression while on cetuximab-based treatment following a complete response, PR or SD, with the development of PD as determined by RECIST version 1.1 within 3 months of the last cetuximab treatment.

### RNA sequencing

RNA was extracted from the macro-dissected tumor portion of the formalin-fixed and paraffin-embedded tissue using TRIzol reagent. A cDNA library was constructed using TruSeq RNA Access Library Prep Kit (Illumina, Inc., San Diego, CA, USA) and paired-end sequencing was conducted using an HiSeq 2500 platform (Illumina Inc.). After sequencing was completed, the raw data were processed with an RNA-S seq analysis pipeline. All fastq format reads were assessed for quality control using FASTQC software (v0.11.8). The Illumina sequencing platform-specific adaptors and poor quality read bases were trimmed using Trim Galore (v.0.4.5). Before the gene expression estimation, the trimmed reads were mapped to the reference genome (human reference genome build version GRCh38/hg38) with STAR aligner (v. 2.6.0) and the output sam/bam files were obtained. The read counts were normalized for effective library size. The Gene Expression Omnibus accession number for the RNA sequencing data is GSE183984.

Differentially expressed genes were analyzed using DESeq2^38^. Differentially expressed genes were defined by an adjusted *P* value < 0.05 and an absolute fold change > 2. Gene set enrichment analysis (GSEA) was performed to calculate the normalized enrichment score (NES)^39^. Immune deconvolution was performed with CIBERSORT to estimate the relative fraction of 22 immune subsets^40^. The enrichment score for the gene signatures was calculated by gene set variation analysis (GSVA)^41^. The RNA sequencing data of the TCGA cohort were obtained from Firebrowse (Broad Institute).

### Multiplexed immunohistochemistry

Optimized fluorescent multiplexed immunohistochemistry was performed using tyramide signal amplification in the Leica Bond Rx Automated Stainer (Leica Biosystems, Newcastle, UK) as previously described^42^. Cells were stained with antibodies against CD68 (M0876, DAKO, Glostrup, Denmark), CD206 (NBP1-90020, Novus Biological, Littleton, CO, USA), PD-L1 (13684S, Abcam, Cambridge, UK) and cytokeratin (NBP2-29429, Novus, Littleton, CO, USA), and the fluorescence signals were captured with the following fluorophores: Opal 570, Opal 620, Opal 690, and Opal 780. Multiplex-stained slides were obtained using the Vectra Polaris Quantitative Pathology Imaging System (Akoya Biosciences, Marlborough, MA/Menlo Park, CA, USA). Regions of interest (ROIs) focusing on the invasive tumor margin or the active tumor-stromal interface were carefully chosen by an experienced pathologist (JK) based on the hematoxylin and eosin slides and cytokeratin expression. The images were analyzed using inForm 2.4.11 image analysis software (Akoya Biosciences, Marlborough, MA/Menlo Park, CA, US) and Spotfire software (TIBCO Software Inc., Palo Alto, CA). The data were expressed as the mean number of cells/mm^2^ for each cell population.

### Statistical analysis

PFS was defined as the interval from the initial date of cetuximab administration (index date) to the date of disease progression (as per RECIST v1.1) or death. The Kaplan–Meier method was used to estimate the survival outcomes. The Mann–Whitney *U*-test was used to compare the non-paired continuous variables. The paired values were compared using the nonparametric Wilcoxon matched-pairs signed-rank test. Correlations between two parameters were evaluated using the Pearson or Spearman correlation coefficient. A *P* value of < 0.05 was considered statistically significant. Statistical analyses were performed using R software version 3.6.2 (R Foundation for Statistical Computing, Vienna, Austria).

## Supplementary Information

Supplementary Information 1.

Supplementary Information 2.

Supplementary Information 3.

Supplementary Information 4.
