# Supplementary material for: Dynamic increase of M2 macrophages is associated with disease progression of colorectal cancers following cetuximab-based treatment
Source: Sci Rep. 2022 Jan 31;12:1678. doi: 10.1038/s41598-022-05694-x (PMC8803829; doi:10.1038/s41598-022-05694-x)
Supplement: Supplementary file 1 — Supplementary Information 1. [file 41598_2022_5694_MOESM1_ESM.docx]

**Supplementary Figure legends**

**Supplementary Figure 1. Progression-free survival of the study patients**

Progression-free survival with cetuximab-based treatments of the whole study population (A), the patient subgroup receiving 1^st^ line treatment (B), and the patient subgroup receiving 3^rd^ line treatment (C).
