# Supplementary material for: Dynamic increase of M2 macrophages is associated with disease progression of colorectal cancers following cetuximab-based treatment
Source: Sci Rep. 2022 Jan 31;12:1678. doi: 10.1038/s41598-022-05694-x (PMC8803829; doi:10.1038/s41598-022-05694-x)
Supplement: Supplementary file 2 — Supplementary Information 2. [file 41598_2022_5694_MOESM2_ESM.pptx]

## Slide 1
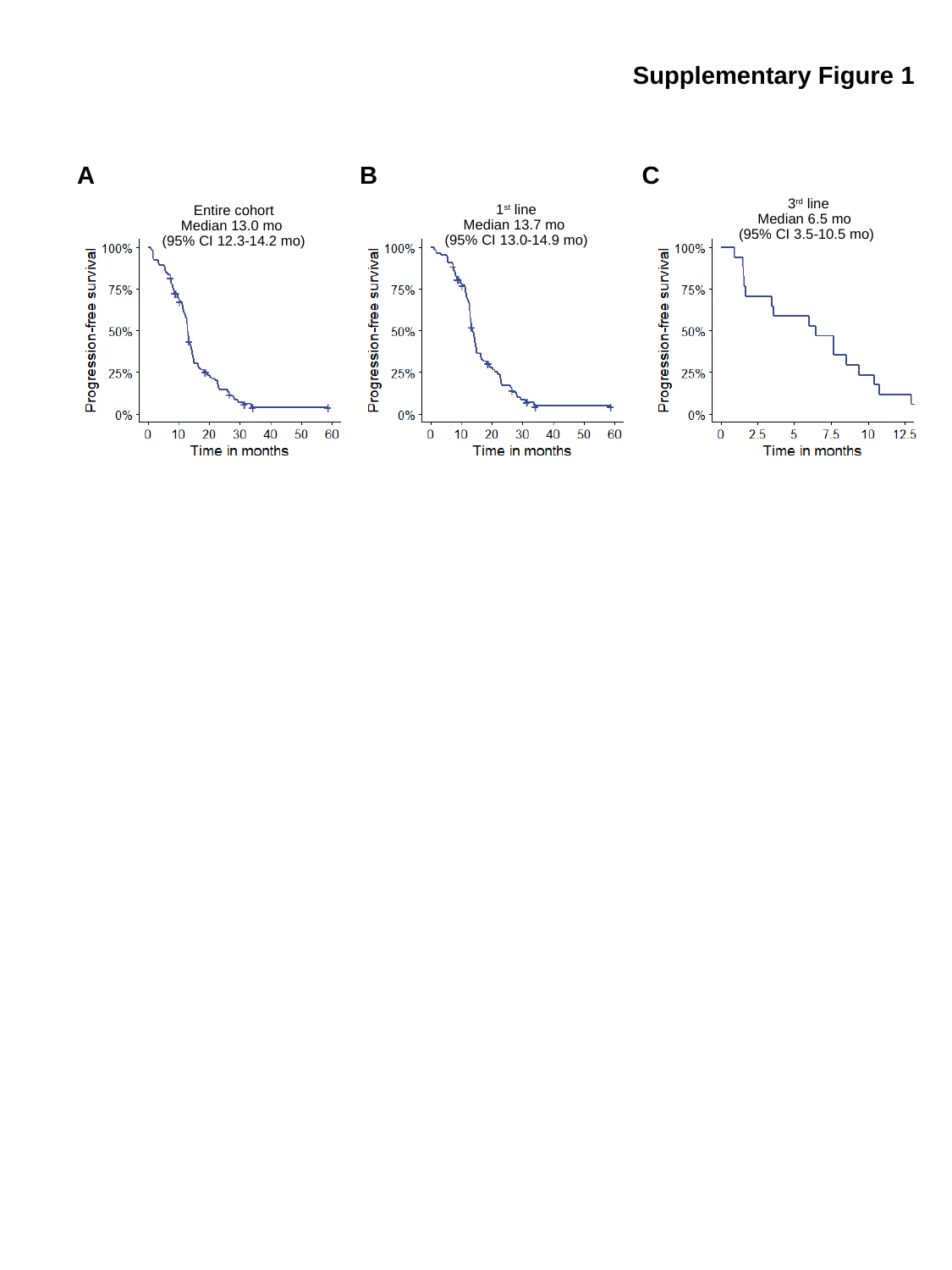

Supplementary Figure 1
A
B
C
 3rd line
Median 6.5 mo
(95% CI 3.5-10.5 mo)
1st line
Median 13.7 mo
(95% CI 13.0-14.9 mo)
Entire cohort
Median 13.0 mo
(95% CI 12.3-14.2 mo)
