# Supplementary material for: Dynamic increase of M2 macrophages is associated with disease progression of colorectal cancers following cetuximab-based treatment
Source: Sci Rep. 2022 Jan 31;12:1678. doi: 10.1038/s41598-022-05694-x (PMC8803829; doi:10.1038/s41598-022-05694-x)
Supplement: Supplementary file 3 — Supplementary Information 3. [file 41598_2022_5694_MOESM3_ESM.docx]

**Supplementary Table 1. Analysis of tumor tissue samples**

| Analysis | Study population (n=106) |
| --- | --- |
| Presence of tumor tissue samples |  |
| Paired samples | 35 (33.0%) |
| Pre-treatment samples only | 55 (51.9%) |
| Post-treatment samples only | 16 (15.1%) |
| RNA sequencing |  |
| Paired samples | 21 (19.8%) |
| Pre-treatment samples only | 55 (51.9%) |
| Post-treatment samples only | 16 (15.1%) |
| Not done | 14 (13.2%) |
| Multiplexed immunohistochemistry | |
| Paired samples | 18 (17.0%) |
| Not done | 106 (83.0%) |
